# Disulfide mapping the voltage-sensing mechanism of a voltage-dependent potassium channel

**DOI:** 10.1038/srep37303

**Published:** 2016-11-17

**Authors:** Tomohiro Nozaki, Shin-ichiro Ozawa, Hitomi Harada, Tomomi Kimura, Masanori Osawa, Ichio Shimada

**Affiliations:** 1 Graduate School of Pharmaceutical Sciences, The University of Tokyo, Hongo, Bunkyo-ku, Tokyo 113-0033, Japan; 2 Faculty of Pharmacy, Keio University, Shibakoen, Minato-ku, Tokyo 105-8512, Japan

## Abstract

Voltage-dependent potassium (Kv) channels allow for the selective permeability of potassium ions in a membrane potential dependent manner, playing crucial roles in neurotransmission and muscle contraction. Kv channel is a tetramer, in which each subunit possesses a voltage-sensing domain (VSD) and a pore domain (PD). Although several lines of evidence indicated that membrane depolarization is sensed as the movement of helix S4 of the VSD, the detailed voltage-sensing mechanism remained elusive, due to the difficulty of structural analyses at resting potential. In this study, we conducted a comprehensive disulfide locking analysis of the VSD using 36 double Cys mutants, in order to identify the proximal residue pairs of the VSD in the presence or absence of a membrane potential. An intramolecular SS-bond was formed between 6 Cys pairs under both polarized and depolarized environment, and one pair only under depolarized environment. The multiple conformations captured by the SS-bond can be divided by two states, up and down, where S4 lies on the extracellular and intracellular sides of the membrane, respectively, with axial rotation of 180°. The transition between these two states is caused by the S4 translocation of 12 Å, enabling allosteric regulation of the gating at the PD.

Voltage-dependent potassium (Kv) channels are membrane proteins that are selectively permeable to potassium ions (K^+^) in a membrane potential dependent manner. Macroscopic current of the Kv channels can be described as a cycle of four stages^1^: (i) no K^+^ current is observed at resting potential (the resting state), (ii) maximum peak current is observed upon depolarization (the activated state), (iii) the current exponentially decays to the constant K^+^ current (the inactivated state), and (iv) the current stops upon repolarization (return to (i)). Through this functional cycle, Kv channels control the action potential, which plays crucial roles in neurotransmission and muscle contraction^1^.

Kv channels are tetrameric proteins, in which each subunit possesses six transmembrane helices, S1-S6. Each subunit consists of a voltage-sensing domain (VSD) that includes S1–S4, and a pore domain (PD) comprised of S5-S6. The center of the tetramer has a K^+^ conducting pore that possesses two gates: a crossing of four S6 helices (helix bundle crossing, HBC) at the intracellular exit of the pore, and a K^+^ selectivity filter (SF) located on the extracellular side of the pore. While the S4 helix of the VSD resides on the intracellular side of the membrane in the resting state, it moves to the extracellular side upon depolarization^2^. This voltage dependent conformational change of the VSD allosterically opens the HBC gate in the PD, leading to channel activation^2^.

To date, structural information at an atomic resolution has been reported for the Kv channels in the absence of a membrane potential. These include the crystal structures of rat Kv1.2^3^,^4^, the chimera of rat Kv1.2 and Kv2.1^5^, and the Kv channels from *Aeropyrum pernix* (KvAP)^6^, as well as a model structure of KvAP^7^. These structures are assumed to represent the activated state, where the S4 helix lies on the extracellular side and the HBC is open. The conformation of the VSD of KvAP is essentially the same as that of the isolated VSD in crystal^6^ and in solution^8^, which is consistent with previous electron paramagnetic resonance (EPR) study showing that the isolated VSD from KvAP in the lipid bilayer retains a very similar conformation to that in the full length KvAP^9^.

However, the structure of the resting state and the voltage-dependent conformational changes have not been determined, because of the difficulty in analyzing the structure at resting potential. Thus, the gating mechanism of the HBC remains elusive.

Several biochemical analyses have revealed the voltage dependent conformational change of VSD. The distance between the membrane surface and each residue of VSD was analyzed by avidin binding to a biotin modified Kv channel, which provided the insights that S3 and S4 move vertical to the membrane, depending on the membrane potential^10^,^11^. Disulfide locking analyses^12^,^13^,^14^ and metal ion bridge analysis^15^ showed the movement distances (6–20 Å) and the rotation angles (30–180°) of S4 upon conformational change. EPR analysis showed that the VSD of KvAP changes its conformation, depending on the lipid environment^16^.

However, there are several problems with these analyses: (1) one or more mutations were introduced to the voltage-sensing Arg residue in S4 and/or its interacting counterpart residues, Asp or Glu, which may modify the voltage-sensing properties of the VSD^12^,^13^,^14^, (2) the conformational change of VSD was detected indirectly, through the change in the K^+^ current of Kv channels^10^,^11^,^12^,^13^,^14^,^15^, (3) the conformational change of VSD was deduced from the results of studying a limited number of mutants^12^,^13^, and (4) the events were investigated during the change of the membrane potential, rather than at a specific potential^10^,^11^,^12^,^13^,^14^,^15^.

In this study, we conducted a comprehensive disulfide locking (SS-locking) analysis of VSD, using 36 double Cys mutants that possess mutations at residues excluding Arg, Asp, and Glu. We identified the proximal Cys residues of VSD by detecting the SS-bond formation in the presence and absence of membrane potential, suggesting the existence of a conformational equilibrium at each membrane potential. Conformational changes of the VSD required for the voltage-sensing is described, based on the results of these analyses.

## Results

In order to analyze the voltage-dependent conformational change of the VSD in liposomes, we first observed the membrane potential of the liposomes reconstituted with VSD. Next, we characterized the voltage dependence of the conformations of the prepared VSD, by observing the fluorescence change of a chemically attached fluorescence moiety. We developed a method for detecting intramolecular SS-bond formation between proximal Cys residues in the double Cys mutant of VSD (SS-locking analysis). This method was applied to 36 double Cys mutants of VSD in liposomes, in the presence and absence of a membrane potential.

### Measurement of the membrane potential on VSD-reconstituted liposomes

Membrane potential on the VSD-reconstituted liposomes was measured based on the fluorescence of 3-(4-(2-(6-(dibutylamino)-2-naphthyl)-*trans*-ethenyl) pyridinium) propanesulfonate (di-4-ANEPPS) (Supplementary Fig. S1), which has reportedly been used for measuring the membrane potentials of empty liposomes and cells^17^.

Di-4-ANEPPS is a membrane potential-sensitive fluorescent dye. Its hydrophobic site is inserted into the membrane while its hydrophilic site is exposed to the aqueous solution. The change in its fluorescence intensity is proportional to the change in the membrane potential. A change in the fluorescence intensity of 9.6% was reported for a 100 mV change in the membrane potential in empty EggPC liposomes, with excitation at 530 nm and emission at 610 nm^17^. Furthermore, in a variety of cells, 7–10% change in the fluorescence intensity was observed when the membrane potential was changed by 100 mV^17^.

In this study, the membrane potential was formed by selective K^+^ influx from high K^+^ buffer (referred to as membrane potential buffer) to the inside of the liposomes with 0.10 mM K^+^. We observed the fluorescence time course for the membrane potential buffer in the presence of di-4-ANEPPS, at various K^+^ concentrations that provide the desired theoretical membrane potential. The 1-palmitoyl-2-oleoyl-sn-glycero-3-phosphoethanolamine (POPE)/1-palmitoyl-2-oleoyl-sn-glycero-3-phospho-(1′-rac-glycerol) (POPG) liposome solution was added to the cuvette at 0 s, and then 1 μL of 40 μM valinomycin, which is a K^+^ selective ionophore, was added at 60 s, to form a membrane potential^18^,^19^.

**Figure 1:**
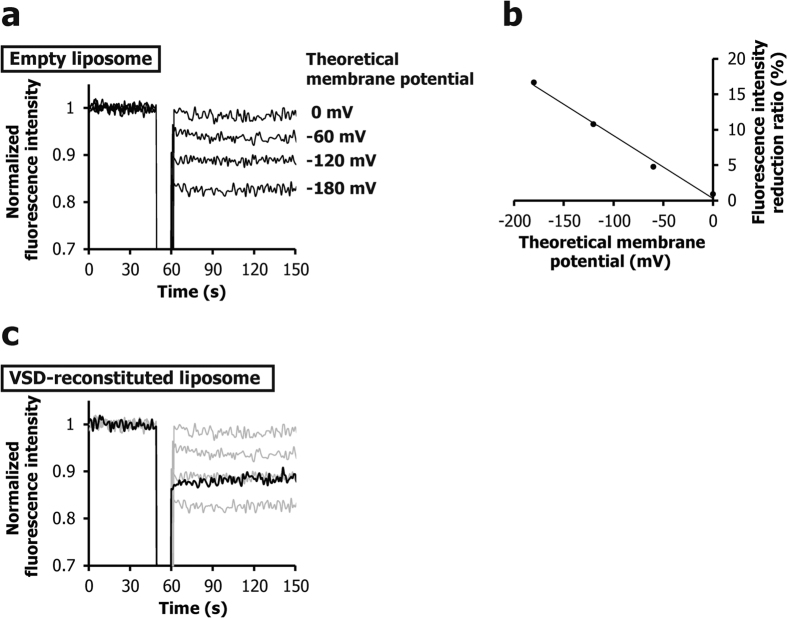
Measurement of membrane potentials of liposomes. (**a**) Voltage dependence of the fluorescence intensity of empty liposomes. (**b**) Relationship between the theoretical membrane potential formed on empty liposomes and the fluorescence intensity reduction ratio of di-4-ANEPPS (panel (a)). (**c**) Fluorescence time-course for the liposomes reconstituted with a VSD mutant, V42C/I131C, where the K^+^ concentration ratio was adjusted to form a −187 mV theoretical membrane potential (black line). The results of (**a**) are shown by gray lines. The potentials corresponded to −140 and −124 mV at a few and 90 s after the addition of valinomycin, respectively.

When the theoretical membrane potentials were 0 mV, −60 mV, −120 mV, and −180 mV, the observed reductions in fluorescence intensity of empty POPE/POPG liposomes were 0.90, 4.8, 10.8, and 16.7%, respectively (Fig. 1a,b). These results indicated that the intensity reductions were proportional to the theoretical values of the membrane potential, and the observed rate of reduction was 0.09% per −1 mV (Fig. 1b). This relationship between the reduction of di-4-ANEPPS fluorescence intensity and the membrane potential was utilized to estimate the membrane potential of numerous VSD-reconstituted POPE/POPG liposomes. Figure 1c shows an example of the estimation of the membrane potential of liposomes reconstituted with a VSD mutant, V42C/I131C. While the theoretical potential was −187 mV, the potentials estimated from the fluorescence intensity reduction were −140 and −124 mV, just after the addition of valinomycin and at 90 s after the addition of valinomycin, respectively. The difference between the theoretical value and the experimental value is probably due to the incomplete K^+^ shield of the liposome membrane reconstituted with the VSD mutant.

### Voltage-dependent conformational change of VSD

**Figure 2:**
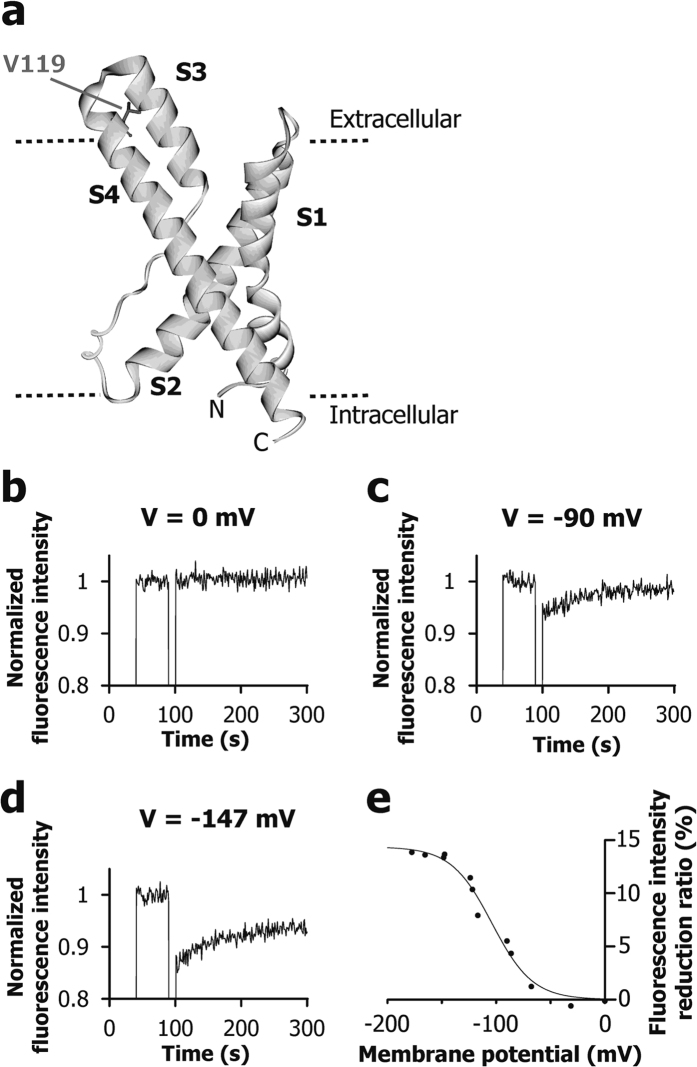
Fluorescence analysis of mBBr to examine voltage-dependent conformational changes of VSD. (**a**) The location of V119, which was mutated to Cys with the mBBr modification. (**b**–**d**) Fluorescence time courses of mBBr attached VSD on the liposomes with three different membrane potentials: (**b**) 0 mV, (**c**) −90 mV, and (**d**) −147 mV. (**e**) Relationship between the estimated membrane potential by di-4-ANEPPS analysis and the fluorescence intensity reduction ratio of mBBr at 100 s (upon the formation of the membrane potential).

In order to investigate the voltage-dependent conformational change of VSD, we used the V119C mutant, in which the Cys119 residue was chemically modified by a fluorescent dye, monobromobimane (mBBr), as previously reported^20^ (Fig. 2a). The conformational change can be detected by the change in the intensity of the fluorescence from mBBr, since the intensity decreases when the fluorophore moves to a more polar environment^21^. The values of membrane potential were estimated for each sample by di-4-ANEPPS analysis.

We prepared POPE/POPG liposomes reconstituted with mBBr-modified VSD. The membrane potential dependent fluorescence changes are shown in Fig. 2b–d. Upon membrane potential formation, instantaneous reduction in fluorescence intensity, followed by time dependent recovery was observed (Fig. 2c,d). The instantaneous intensity reduction indicates that the fluorophore at Cys119 moves to a more polar environment. The reduction ratios of the fluorescence intensity depended on the membrane potential, indicating that the VSD on the liposome experiences a voltage-dependent conformational change. Sigmoidal curve fitting of the fluorescence intensity reductions against the membrane potential revealed that the activation potential (V_1/2_) of VSD was −104 mV (Fig. 2e). It should be noted that time dependent recovery of fluorescence intensity (Fig. 2c,d) also reflects the conformational change of the VSD, corresponding to the decay of membrane potential (Fig. 1c). For example, in Fig. 2d, the intensity reduction ratios were 13.7% and 6.6% at 100 s (upon the formation of the membrane potential) and 300 s (200 s after the formation of the membrane potential), respectively. The membrane potentials estimated by di-4-ANEPPS analysis were −147 mV upon the formation of the membrane potential and −116 mV in 200 s (data not shown), respectively. The relationship between the membrane potential of −116 mV and the intensity reduction of the mBBr fluorescence by 6.6% at 200 s after the membrane potential formation (Fig. 2d), is consistent with the sigmoidal curve shown in Fig. 2e, which was constructed from a number of experiments with different membrane potential values upon the membrane potential formation.

The activation potential, which is lower by about 40 mV than that obtained from the G-V curve of full length KvAP^22^, is consistent with the reports that the activation potential of VSD is lower than that of Kv channels, in analyses of KvAP and other Kv channels^23^,^24^,^25^. Therefore, the voltage-dependent conformational change in the VSDs used in this study is likely to be essentially the same as that in the full length KvAP.

### Detection of an intramolecular SS-bond in VSD double Cys mutants

**Figure 3:**
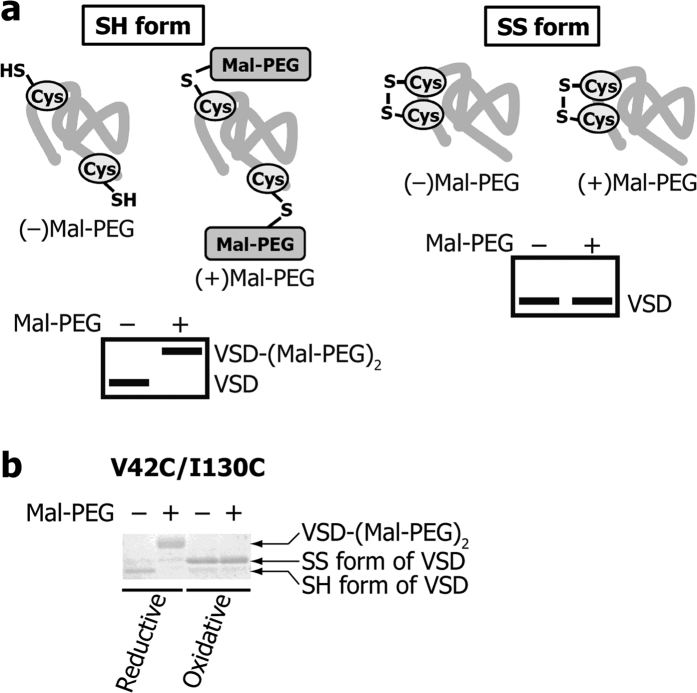
Detection of an intramolecular SS-bond. (**a**) Schematic diagram of the intramolecular SS-bond detection. The SH form of the VSD mutant is PEGylated and exhibited a significant mobility shift (left), while the SS form of the VSD mutant is not PEGylated, leaving the mobility unchanged (right). (**b**) SDS-PAGE mobility of the VSD double Cys mutant (V42C/I130C) under reductive or oxidative conditions in the presence or absence of mal-PEG.

The free thiol groups in a protein can be modified by maleimide polyethylene glycol (mal-PEG). The modification can be detected as a mobility shift in the SDS-PAGE analysis^26^,^27^. In order to estimate the intramolecular SS-bond formation in the VSD double Cys mutants by detecting free thiol groups, VSD double Cys mutants, which were pre-denatured in SDS and urea, were treated with mal-PEG with a molecular weight of ca. 2,000 (Fig. 3a).

First, a double Cys mutant (V42C/I130C) in n-decyl-β-D-maltopyranoside (DM) detergent micelles was denatured after reduction or oxidation, and then treated with mal-PEG. For the reduced VSD double Cys mutant, the mal-PEG treatment caused mobility shift in SDS-PAGE, and the band without the PEG modification (PEGylation) disappeared (Fig. 3b, Reductive), indicating that the two thiol groups were PEGylated and no SS-bond was formed between the two Cys residues. However, for the oxidized VSD double Cys mutant, a band without PEGylation was observed (Fig. 3b, Oxidative), indicating that the two Cys residues formed an SS-bond that precluded PEGylation. The SS-bond formation between C42 and C130 caused slight mobility shift in SDS-PAGE, compared to that of the SH form, since the molecular shape of VSD in the SDS micelles is modified by the intramolecular SS-bond. However, the difference in the mobility between the SS and SH forms depends on the Cys positions, and in some cases, no significant difference in the mobility was observed. PEGylation used in this study helped the detection of SH form clearly. These results also indicated that the reaction of the mal-PEG modification proceeds faster than the intramolecular SS-bond formation between the free thiol groups in the denaturation buffer.

**Figure 4:**
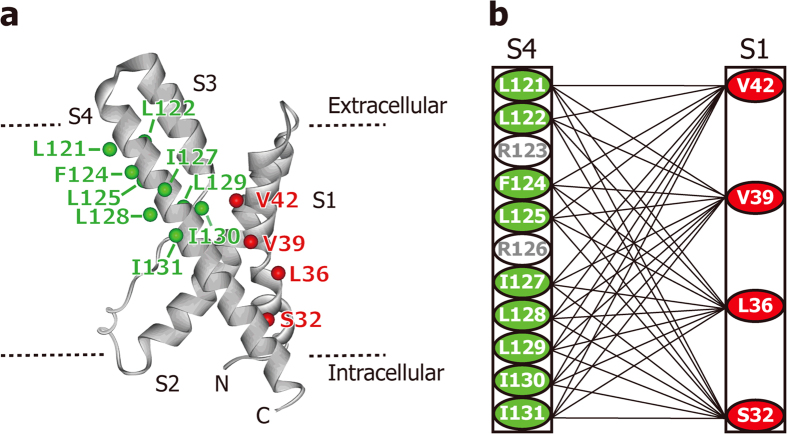
Locations of Cys mutation on VSD. (**a**) The locations of the residues mutated to Cys are shown as spheres (residues on S1 and S4 are colored red and green, respectively) on the VSD crystal structure (PDB code 1ORS). (**b**) The analyzed residue pairs are connected by lines.

Next, we analyzed the intramolecular SS-bond formation of the VSD double Cys mutants reconstituted in liposomes, in the presence or absence of a membrane potential. Since the negative membrane potential was applied to the outside of the liposomes (Supplementary Fig. S1), the N and C termini of the VSD needed to be on the outer surface of the liposomes. In order to analyze the VSD that is exposed to membrane potential in the right direction, we reconstituted the maltose binding protein (MBP)-fused VSD in liposomes and digested the MBP outside the liposomes by human rhinovirus (HRV) 3C protease. This procedure enables the discrimination of the VSD with an MBP-tag outside of the liposomes from that with the tag inside, in SDS-PAGE, since the latter possesses higher molecular weight because of the uncleaved MBP (Supplementary Fig. S2). The digested VSD was analyzed by SS-locking after negative membrane potential was created outside the liposomes by K^+^ influx. We applied this method to the 36 VSD double Cys mutants (Fig. 4).

### Voltage-dependent SS-locking analysis of 36 VSD double Cys mutants

In order to comprehensively search for the residue pairs that are proximate to each other at resting potential, we prepared 36 double Cys mutants, in which mutations were introduced at one of 4 residues on S1 (residues located on the S4 side; S32, L36, V39, or V42), and one of 9 residues on S4 (from L121 to I131 except for Arg; L121, L122, F124, L125, I127, L128, L129, I130, or I131) (Fig. 4). It should be noted that the conformation of S4 and not that of S1, is assumed to change drastically depending on the membrane potential.

Liposomes reconstituted with a VSD double Cys mutant were diluted with the membrane potential buffer containing valinomycin, in which the K^+^ concentrations were the same as and 1000-fold higher than that on the inside of the liposomes, to create theoretical membrane potentials of 0 and −187 mV, respectively. The values of membrane potential were estimated for each sample by the di-4-ANEPPS analysis. These VSD double Cys mutants were incubated in the presence of 30 μM Cu^2+^-o-phenanthroline (CuP) for 1 min at room temperature (25 °C), to allow intramolecular SS-bond formation, between two Cys residues that are in close proximity of each other (when two Cys residues form an SS-bond, the distance between the two Cβ atoms of the Cys residues is within 4.6 Å^28^). The incubation for 1 min was the shortest period needed for handling the samples in the experiments. The temperature was set to the same as that for the fluorescence experiments to evaluate membrane potential. The oxidative effect of CuP was quenched after 1 min by 5 mM ethylenediaminetetraacetic acid (EDTA). After the removal of the liposomal lipids, the VSD mutants were denatured with SDS and urea, treated simultaneously with mal-PEG, and then analyzed by SDS-PAGE. An unPEGylated band provides good evidence of the SS-bond formation that protects the protein from the PEGylation. The SS-bond formation was judged as “+”, when the intensity of the unPEGylated band is higher than 25% of the total intensity of the PEGylated and unPEGylated bands. It should be noted that this analysis could not discriminate between transitions with high and low probability, since the SS-bond is formed even at a single occurrence of close proximity. Therefore, we did not analyze the extent of the SS-bond formation quantitatively, but evaluated qualitatively the possibility of two Cys residues coming into close proximity.

**Figure 5:**
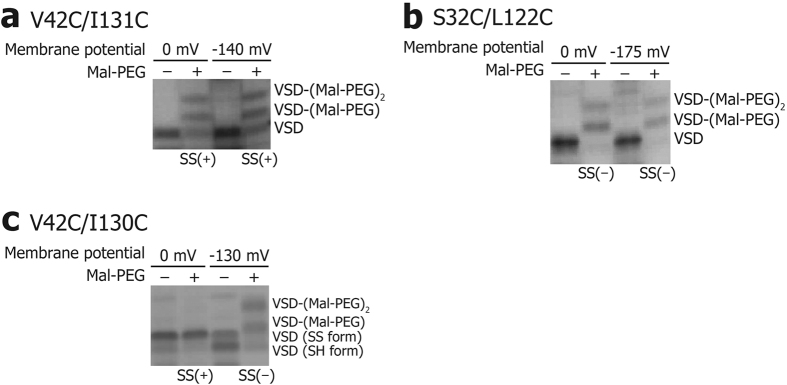
Voltage-dependent SS-locking analysis. The SS-locking analysis results of three VSD double Cys mutants are shown. SS(+) stands for the SS-bond formation, as evidenced by the intensity of the unPEGylated band higher than 25% of the total intensity of the PEGylated and unPEGylated bands. SS(−) stands for no SS-bond formation, as evidenced by the intensity of the unPEGylated band lower than 25% of the total intensity of the PEGylated and unPEGylated bands. (**a**) V42C/I131C, (**b**) S32C/L122C, and (**c**) V42C/I130C.

Three representative results of the SS-locking analysis are shown in Fig. 5. In Fig. 5a, for the V42C/I131C mutant, an SDS-PAGE band for an unPEGylated VSD was observed at 0 and −140 mV, even with mal-PEG treatment. In contrast, in Fig. 5b, for the S32C/L122C mutant, the SDS-PAGE band corresponding to unPEGylated protein disappeared with the mal-PEG treatment, and two bands corresponding to PEGylated protein were observed at both 0 and −175 mV, indicating that one or two Cys residues are subject to the PEGylation. Considering the fact that the unPEGylated band disappeared with the mal-PEG treatment in Fig. 5b, the first PEGylation for one of the free thiol groups proceeds sufficiently quickly, while the second PEGylation seems slower than the first one. Since the molecular weight of mal-PEG is ca. 2,000, steric hindrance between a VSD-attached mal-PEG molecule and a free mal-PEG could reduce the reaction rate of the second mal-PEG molecule, even when the VSD is denatured. Indeed, at least one mal-PEG molecule is bound to all the mutants as shown in Fig. 5 and Supplementary Figs S3–6. The relative intensity ratio of the bands for the VSD – Mal-PEG molecule and the VSD – (Mal-PEG)_2_ molecule varied, depending on the positions of the Cys residues.

A comparison of the results in Fig. 5a,b revealed that not all, but a significant amount, of the V42C/I131C mutant formed an SS-bond, which protected the protein from PEGylation, and that no SS-bond was formed in the V32C/L122C mutant between the two thiol groups of the Cys residues. Thus, in the wild type VSD, V42 and I131 would approach each other at 0 and −140 mV, while S32 and L122 do not, at either 0 or −175 mV.

In Fig. 5c, for the V42C/I130C mutant, an unPEGylated band was observed only at 0 mV (depolarized), indicating that SS-bonds were formed between V42C and I130C, under the depolarized conditions. Thus, these results suggested that V42 and I130 could approach each other only under depolarized conditions. While V42C/I130C exhibited an unPEGylated band without the mal-PEG treatment (Fig. 5c), the SS-bond was expected to be formed not during the incubation period at the membrane potential of −130 mV, but under the denatured condition afterwards, because the sample with mal-PEG treatment did not show unPEGylated band.

We next investigated whether each of the other 32 double Cys mutants could form an SS-bond under the depolarized and/or polarized conditions, based on the existence of the unPEGylated band in SDS-PAGE (Supplementary Figs S3–S6 and summarized in Supplementary Table S1).

**Figure 6:**
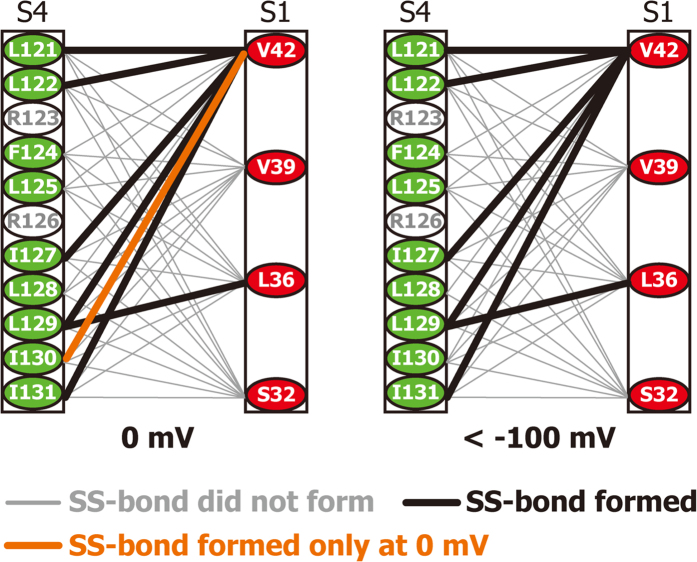
Schematic diagram of the results of the voltage-dependent SS-locking analysis. Black bold lines show residue pairs that formed an SS-bond. The orange line shows the residue pair, which only formed an SS-bond at 0 mV.

The results are schematically summarized in Fig. 6. Under the depolarized conditions (0 mV), the intramolecular SS-bond formation was observed for 7 mutants L36C/L129C, V42C/L121C, V42C/L122C, V42C/I127C, V42C/L129C, V42C/I130C, and V42C/I131C. Under the polarized conditions (<−100 mV), the intramolecular SS-bond formation was observed for 6 mutants L36C/L129C, V42C/L121C, V42C/L122C, V42C/I127C, V42C/L129C, and V42C/I131C.

The unPEGylated band might be due to oxidation of a thiol group to a sulfino or sulfonic group without the formation of the intramolecular SS-bond. In order to investigate whether the PEGylation was protected by the SS-bond or not, we reduced SS-bonds after CuP oxidation, followed by mal-PEG modification (Supplementary Fig. S7). Reduced samples did not show unPEGylated bands, but showed the PEGylated bands. These results clearly showed that these mutants formed intramolecular SS-bond and were not oxidized to a sulfino or sulfonic group.

Among these mutants, all but one residue pair formed an SS-bond at both 0 and <−100 mV (bold black lines in Fig. 6 connecting the two Cys residues). In particular, the residues L121C and I131C, which formed an SS-bond with V42C both at 0 and <−100 mV, are 10-residues apart on the S4 helix. If a single conformation of VSD is assumed, it would be impossible for L121 and I131 to simultaneously approach V42, strongly suggesting that the VSD adopts multiple conformations at both membrane potentials.

Only one mutant, V42C/I130C, exhibited significant differences in the SS-bond formation between 0 mV and <−100 mV: an SS-bond was formed at 0 mV (orange line in Fig. 6), and not at −130 mV.

Considering the fact that the VSD adopts multiple conformations under the polarized and depolarized conditions, the voltage-dependent difference in the SS-bond formation suggests that the multiple conformations are differently populated under the polarized and depolarized conditions.

## Discussion

In this study, we analyzed the conformations of the isolated VSD in the lipid bilayer in the presence or absence of a membrane potential. Previous studies indicate that the isolated VSD from KvAP in the lipid bilayer adopts a similar conformation to that from the full length KvAP^9^. Consistent with this finding, our fluorescence analysis indicated that the sigmoidal relationship between the membrane potential and the conformational change of the VSD (Fig. 2e) is also similar to that of the full length KvAP. Time dependent recovery of fluorescence intensity after instantaneous reduction (Fig. 2c,d) seems to correspond to the decay of membrane potential (Fig. 1c).

In order to detect proximal residue pairs of the VSD, we conducted voltage-dependent SS-locking analyses for VSD possessing double Cys mutations in one of four residues in S1 (S32, L36, V39, V42) and one of nine residues in S4 (L121 - I131 except the two Arg residues) (Fig. 4). The residue pairs that formed an SS-bond at 0 and <−100 mV are summarized in Fig. 6. While one residue pair exhibited voltage-dependent difference in the SS-bond formation (orange), six residue pairs formed SS-bonds at both membrane potentials (bold black lines). In particular, V42C, located on the extracellular side of the S1 helix, formed an SS-bond with the S4 residues at the N-terminal end (L121C and L122C) and those at the C-terminal end (I127C, L129C, and I131C), while no SS-bond was observed for V42C with F124C or L125C, located in the middle of S4. These results strongly suggest that the S4 helix can exist stably in the intracellular (down) and extracellular (up) positions, where the N and C-terminal ends of S4 are close to the extracellular end of S1, respectively, and that the S4 helix is unable to remain in the middle position.

**Figure 7:**
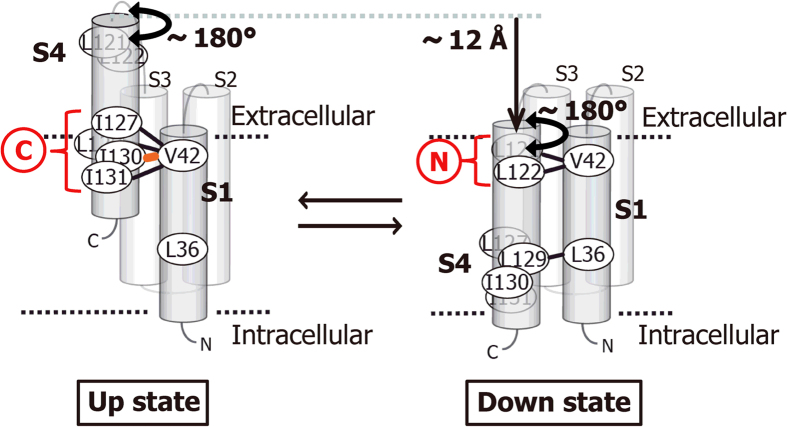
Conformational equilibrium of the VSD. VSD exists in an equilibrium between the up and down states in either polarized or depolarized environment.

Assuming the two states where the S4 helix resides in the up and down positions (referred to as the up and down states, respectively, Fig. 7), all of the residue pairs forming an SS-bond are close to each other in either state. In the up state, V42 in S1 is close to I127, L129, I130, and I131 in S4 (Fig. 7, left). In the down state, V42 is close to L121 and L122 in S4, and at the same time, L36 in S1 is close to I129 in S4 (Fig. 7, right). In each state, however, 2–3 continuous residues in S4 formed an SS-bond with one of the residues in S1, indicating that the S4 helix possesses flexibility with an axial rotation of about 180°. These results suggest that each state is not composed of a single conformation, but rather contains an ensemble of multiple conformations. Since the residue pairs connected by bold black lines in Figs 6 and 7 formed SS-bonds at both membrane potentials, the VSD seems to exist in a conformational equilibrium between the up and down states, where each state is also in equilibrium among multiple conformations.

While six Cys residue pairs formed an SS-bond regardless of the magnitude of the membrane potential, the SS-bond between V42C-I130C was observed only at 0 mV (orange in Figs 6 and 7). Assuming that the VSD adopts either of the up or down state, this result suggests that depolarization of the membrane increases the proportion of the up state.

L129C forms an SS-bond with V42C in the up state and with L36C in the down state. Since the Cβ atoms of L36 and V42 are 12 Å apart, the distance of the S4 translational shift is estimated at about 12 Å (Fig. 7). Therefore, these results suggest that changes of the membrane potential are sensed by the S4 translocation that are 12 Å at the largest, leading to the allosteric regulation of the gating at the PD^2^,^29^.

Previous studies also detected voltage-dependent conformational changes^10^,^11^,^12^,^13^,^14^,^15^ and lipid-driven conformational change^16^ of the VSD. The voltage-dependent conformational change suggested from this study seems to be different from the lipid-driven conformational change^16^. We assume that the down state in Fig. 7 is different from the conformation in non-phospholipid bilayer. However, we cannot compare the conformations of VSD precisely, because we cannot acquire detailed structural data of VSD in non-phospholipid bilayer.

On the other hand, our estimation of the S4 translocation is similar to, but not completely the same as those in the previously reported values ranging from 6 to 20 Å, which were obtained by different methodologies such as other disulfide locking analyses^12^,^13^,^14^ and a metal-ion bridge analysis^15^. It should be noted that, in the previous reports, Cys mutations were introduced at charged residues such as Arg, Asp, or Glu^12^,^13^,^14^,^15^, although the membrane potential is sensed by the Arg residues that interact with Asp/Glu residues. In this study, we avoided introducing mutations at these key residues to preserve the electrophysiological properties of VSD, since mutation of these residues may modify the mode of the voltage-dependent conformational change of VSD.

## Methods

### Expression and purification of VSD

The DNA encoding Met -12 to Lys 147 of the KvAP channel, with an N-terminal decahistidine tag, followed by an HRV 3C protease cleavage site, was inserted into the pMAL-c2X plasmid (New England Biolabs). To prevent the artificial dimerization of VSD, Cys -2 was substituted with Ser by the QuikChange^®^ system (Strategene). Hereafter, this mutant, Cys-2Ser, is referred to as VSD. The site specific Cys mutations described below, were also introduced into the VSD by QuikChange^®^. We prepared the V119C mutant for fluorescent labeling, and 36 double Cys mutants for SS-locking (see below), in which we mutated one residue in S1 (S32, L36, V39, or V42) and another one in S4 (L121, L122, F124, L125, I127, L128, L129, I130, or I131) to Cys.

The VSD and its mutants were expressed in *E. coli* XL1-Blue cells, as fusion proteins with an N-terminal MBP and a decahistidine tag (hereafter, the expressed proteins are referred to as MBP-VSD). The cells were grown at 37 °C to an A_600_ of 0.4–0.6, and protein expression was then induced with 1.0 mM isopropyl-β-D-thiogalactopyranoside for 3–6 hours at 37 °C. Purification of the MBP-VSD was described elsewhere^20^. For the reconstitution of the purified proteins, the buffer was exchanged with “liposome buffer”, containing 20 mM Hepes-NaOH, pH 8.0, 0.10 mM KCl, 149.9 mM NaCl, and 10%(v/v) glycerol, in the presence of 2 mM DM. The purified MBP-VSDs were concentrated to 100–200 μM and stored at 4 °C, after addition of 10 mM dithiothreitol (DTT).

### Preparation of liposomes

A 3:1 (w/w) mixture of POPE/POPG (Avanti) was dried under nitrogen stream and then suspended at a concentration of 10 mg/mL in liposome buffer containing 100 mM DM. For the preparation of empty liposomes, the lipid suspension was slowly rotated at room temperature for 3 hours. For the preparation of VSD-reconstituted liposomes, the lipid suspension was rotated at room temperature for 1 hour, followed by the addition of the MBP-VSD at a weight ratio of 1:200 for VSD/lipid, and further rotation at room temperature for 2–3 hours. Then, the solution was dialyzed against a 500–1000 times volume of the liposome buffer with a 10,000 MWCO membrane (Spectrum), at 4 °C for 3–5 days. After dialysis, the liposomal solution was extruded on an Avanti^®^ Mini-Extruder (Avanti), with 11 passes through a 0.1 μm Nuclepore membrane (Whatman). The MBP residing on the outside of the liposomes was removed by digesting the linker between the MBP and the VSD with HRV 3C protease (Novagen).

### Measurement of the membrane potential of the liposomes

The prepared liposomes were diluted with “membrane potential buffer”, containing 20 mM Hepes-NaOH, pH 8.0, x mM KCl (x = 0.1–150), 150–x mM NaCl, and 10% (v/v) glycerol, which is the same as the liposome buffer except for the concentrations of KCl and NaCl. Then, a membrane potential was formed on the liposome membrane by the addition of valinomycin, a K^+^ selective ionophore, in DMSO solution.

We measured the membrane potential of the liposomes by observing the change in the fluorescence intensity of the membrane potential sensitive fluorescent dye, di-4-ANEPPS. Data were collected at 25 °C on an RF-5300PC spectrofluorometer (Shimadzu).

Briefly, 10 μL of 100 μM di-4-ANEPPS DMSO solution and 50 μL liposome buffer were added to 1700 μL membrane potential buffer (see Table 1 for the concentrations of KCl and NaCl in the membrane potential buffer), and then fluorescence time course measurement was started with excitation at 530 nm and emission at 610 nm. At 0 s, a 40 μL aliquot of liposomes was added, and after a 60 s interval, 1 μL of 40 μM valinomycin was added to form a membrane potential. The fluorescence intensity following the addition of valinomycin was estimated from the linear regression of the fluorescence intensity for 45 s.

### Fluorescence analysis of mBBr-modified VSD

Monobromobimane-modified VSD was prepared according to the published procedure^20^. The fluorescence analysis was conducted with membrane potential buffer (Table 1).

The fluorescence time course was observed for 1800 μL membrane potential buffer, with excitation at 394 nm and emission at 470 nm. At 30 s, a 40 μL aliquot of liposomes was added, and 1 μL of 40 μM valinomycin was added at 90 s, to form a membrane potential. The fluorescence intensity upon the formation of the membrane potential by the addition of valinomycin (at 100 s) was estimated from the linear regression of the fluorescence intensity for the following 45 s. The values of membrane potential were estimated for each sample by di-4-ANEPPS analysis, as shown in “Measurement of the membrane potential of the liposomes”.

In order to obtain the relationship between the membrane potential and the fluorescence intensity reduction ratio, we conducted this analysis with identical liposome sample and different membrane potential buffers whose theoretical membrane potentials are 0, 40, 80, 100, 110, 120, 130, 140, 150, 160, 180, and 188 mV.

### Detection of an intramolecular SS-bond in the VSD in DM micelle

The VSD double Cys mutants were incubated with either 10 mM DTT as a reductant or 100 μM CuP as an oxidant at room temperature for 1 hour. The CuP was inactivated by the addition of 500 μM EDTA.

The VSD was collected as a precipitate by chloroform/methanol extraction^30^. The VSD precipitate was solubilized in “denaturation buffer” (15 mM Tris-HCl, pH 7.4, 2% (w/v) SDS, 6 M urea) containing 0 mM or 1 mM mal-PEG, with a molecular weight of about 2,000 (SUNBRIGHT^®^ ME-20MA (NOF)), incubated at room temperature for 3 hours, and analyzed by SDS-PAGE.

### Voltage-dependent SS-locking analysis

Liposomes reconstituted with VSD double Cys mutant were prepared as described above, with 1 mM DTT. The DTT in the liposome solution was removed by gel filtration with PD miditrap^TM^ G-25 (GE Healthcare). It should be noted that MBP-VSD is digested by HRV 3C protease when the MBP resides on the outside of the liposome, and MBP on the inside of the liposome remains fused with the VSD. The digested MBP and the HRV 3C protease, both of which were His-tagged, were removed by His-select^®^ Nickel affinity gel (SIGMA-ALDRICH).

For the oxidative SS-bond formation, 760 μL membrane potential buffer (KCl concentration 0.10 mM or 150 mM), 1 μL of 40 μM valinomycin, 40 μL liposome solution, and 8 μL of 3 mM CuP (final concentration at 30 μM) were mixed on a shaking vortex mixer at room temperature. The oxidation was stopped with 5 mM EDTA at 1 min. In order to oxidize only at the polarized membrane potential, oxidation was conducted for 1 min, during which, about 8% of the membrane potential decayed (Fig. 1c). The values of membrane potential were estimated for each sample by di-4-ANEPPS analysis, as shown in “Measurement of the membrane potential of the liposomes”.

Next, the VSD was extracted as a precipitate with chloroform/methanol^30^, solubilized with denaturation buffer containing 0 mM or 1 mM mal-PEG, and incubated at room temperature overnight, and analyzed by SDS-PAGE. An intramolecular SS-bond was detected by the band without PEGylation, which is observed only when the two thiol groups formed the intramolecular SS-bond (n = 1–2).

### Confirmation of the SS-bond formation

Liposomes reconstituted with VSD double Cys mutant were prepared as described above, with 1 mM DTT. The MBP-tag on the outside of the liposome was digested by HRV 3C protease. The DTT in the liposome solution was removed by gel filtration with PD miditrapTM G-25. 380 μL liposome buffer, 20 μL liposome solution, and 4 μL of 3 mM CuP (final concentration at 30 μM) were mixed on a shaking vortex mixer and incubated for 1 hour at room temperature. The oxidation was stopped with 5 mM EDTA.

Next, the VSD was extracted as a precipitate with chloroform/methanol, chloroform/methanol extraction was conducted twice, solubilized with liposome buffer containing 40 mM DM and 0 mM or 10 mM tris(2-carboxyethyl)phosphine (TCEP), and incubated at room temperature for 1 hour. VSD was extracted as a precipitate with chloroform/methanol, chloroform/methanol extraction was conducted twice, solubilized with denaturation buffer containing 0 mM or 1 mM mal-PEG, and incubated at room temperature overnight, and analyzed by SDS-PAGE.

## Additional Information

**How to cite this article**: Nozaki, T. *et al.* Disulfide mapping the voltage-sensing mechanism of a voltage-dependent potassium channel. *Sci. Rep.* **6**, 37303; doi: 10.1038/srep37303 (2016).

**Publisher’s note**: Springer Nature remains neutral with regard to jurisdictional claims in published maps and institutional affiliations.

## Supplementary Material

Supplementary Information

## Figures and Tables

**Table 1:** Concentrations of KCl and NaCl in the membrane potential buffer.

Theoretical membrane potential (mV)	187	180	160	150	140	130	120
KCl (mM)	150.0	110.3	50.6	34.3	23.3	15.8	10.7
NaCl (mM)	0	39.7	99.4	115.7	126.8	134.2	139.3
**Theoretical membrane potential (mV)**	**110**	**100**	**80**	**60**	**40**	**20**	**0**
KCl (mM)	7.2	4.9	2.3	1.0	0.5	0.2	0.1
NaCl (mM)	142.8	145.1	147.8	149.0	149.5	149.8	149.9
