# Supplementary material for: Disulfide mapping the voltage-sensing mechanism of a voltage-dependent potassium channel
Source: Sci Rep. 2016 Nov 17;6:37303. doi: 10.1038/srep37303 (PMC5112519; doi:10.1038/srep37303)
Supplement: Supplementary Information [file srep37303-s1.pdf]

Disulfide mapping the voltage-sensing mechanism of a voltage-dependent potassium channel

Tomohiro Nozaki[1], Shin-ichiro Ozawa[1], Hitomi Harada[1], Tomomi Kimura[1], Masanori Osawa[1,2,*], and Ichio Shimada[1,*]

[1]Graduate School of Pharmaceutical Sciences, The University of Tokyo, Hongo, Bunkyo-ku, Tokyo 113-0033, Japan

[2]Faculty of Pharmacy, Keio University, Shibakoen, Minato-ku, Tokyo 105-8512, Japan

*Correspondence to osawa-ms@pha.keio.ac.jp and shimada@iw-nmr.f.u-tokyo.ac.jp

Supplementary Table S1: Summary of the voltage-dependent SS-locking results for 36 double Cys mutants of VSD

The membrane potential values, estimated by using di4-ANNEPS, are shown in the upper row, and the SS-bond formation is shown in the lower row, for each double mutant.

"+" stands for the SS-bond formation, as evidenced by the intensity of the unPEGylated band higher than 25% of the total intensity of the PEGylated and unPEGylated bands.

"-" stands for no SS-bond formation, as evidenced by the intensity of the unPEGylated band lower than 25% of the total intensity of the PEGylated and unPEGylated bands.

N.D. : Neither VSD nor a PEGylated VSD band was detected in the SDS-PAGE analysis. These data were excluded from the analysis.

		Mutagenesis position (S1)							
		S32		L36		V39		V42	
Mutagenesis position (S4)	L121	0 mV	-177 mV	0 mV	-105 mV	0 mV	-122 mV	0 mV	-178 mV
		-	-	-	-	-	-	+	+
	L122	0 mV	-175 mV	0 mV	-175 mV	0 mV	-114 mV	0 mV	-163 mV
		-	-	-	-	-	-	+	+
	F124	0 mV	-158 mV	0 mV	-134 mV	0 mV	-130 mV	0 mV	-114 mV
		-	N.D.	-	-	-	-	-	-
	L125	0 mV	-160 mV	0 mV	-153 mV	0 mV	-116 mV	0 mV	-141 mV
		-	-	-	-	-	-	-	-
	I127	0 mV	-163 mV	0 mV	-162 mV	0 mV	-125 mV	0 mV	-149 mV
		-	-	-	-	-	-	+	+
	L128	0 mV	-164 mV	0 mV	-157 mV	0 mV	-117 mV	0 mV	-136 mV
		-	-	-	-	-	-	-	-
	L129	0 mV	-144 mV	0 mV	-153 mV	0 mV	-106 mV	0 mV	-128 mV
		-	-	+	+	-	-	+	+
	I130	0 mV	-160 mV	0 mV	-140 mV	0 mV	-101 mV	0 mV	-130 mV
		-	-	-	-	-	-	+	-
	I131	0 mV	-181 mV	0 mV	-145 mV	0 mV	-102 mV	0 mV	-140 mV
		-	-	-	-	-	-	+	+

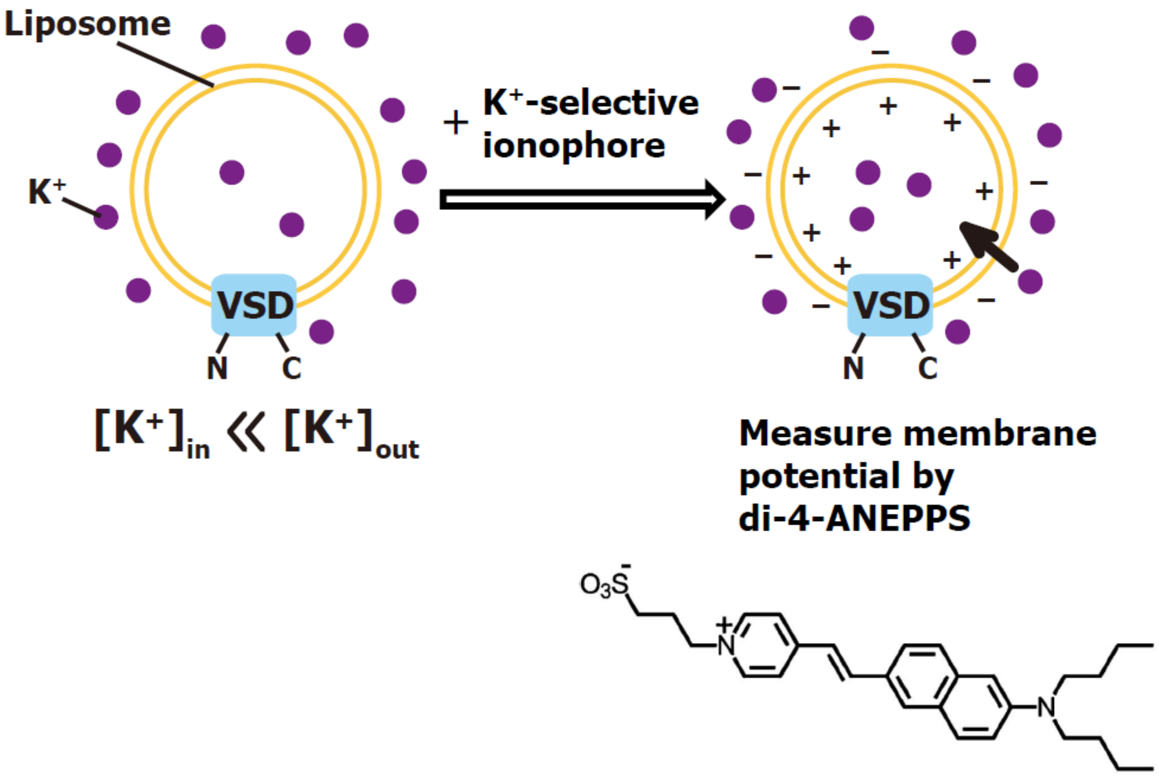

Supplementary Figure S1: Schematic diagram of membrane potential formation and the structure of di-4-ANEPPS

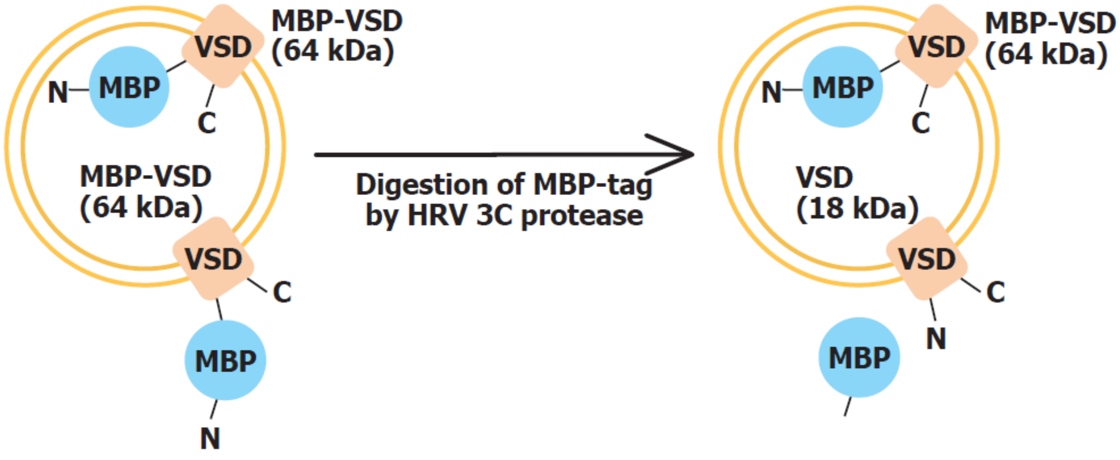

Supplementary Figure S2: Schematic diagram of the method to analyze the orientation of VSD

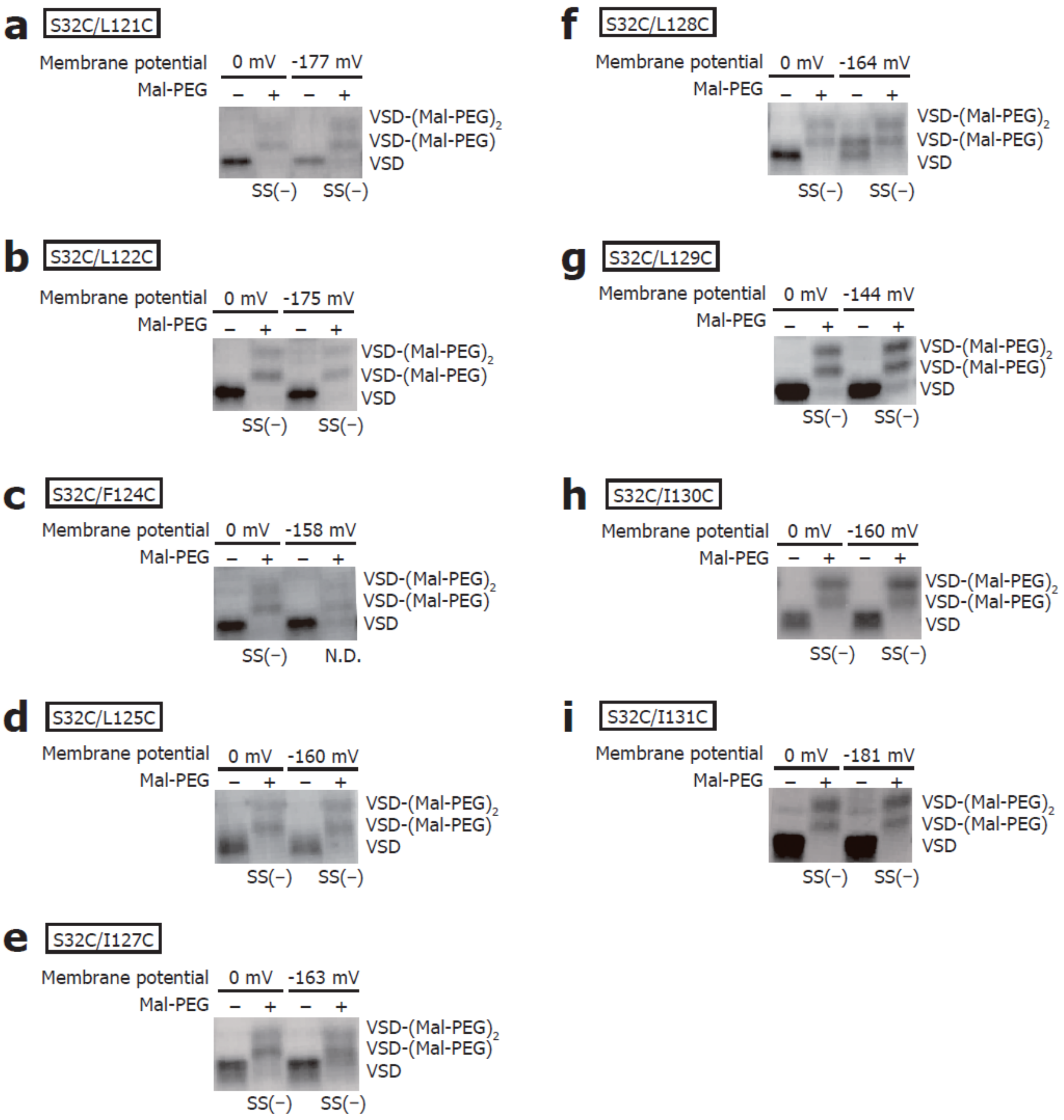

Supplementary Figure S3: The results of the voltage-dependent SS-locking analysis (S32 in S1 was mutated to Cys)

SS(+) stands for the SS-bond formation, as evidenced by the intensity of the unPEGylated band higher than 25% of the total intensity of the PEGylated and unPEGylated bands.

SS(-) stands for no SS-bond formation, as evidenced by the intensity of the unPEGylated band lower than 25% of the total intensity of the PEGylated and unPEGylated bands.

N.D. : Neither VSD nor a PEGylated VSD band was detected in the SDS-PAGE analysis. These data were excluded from the analysis.

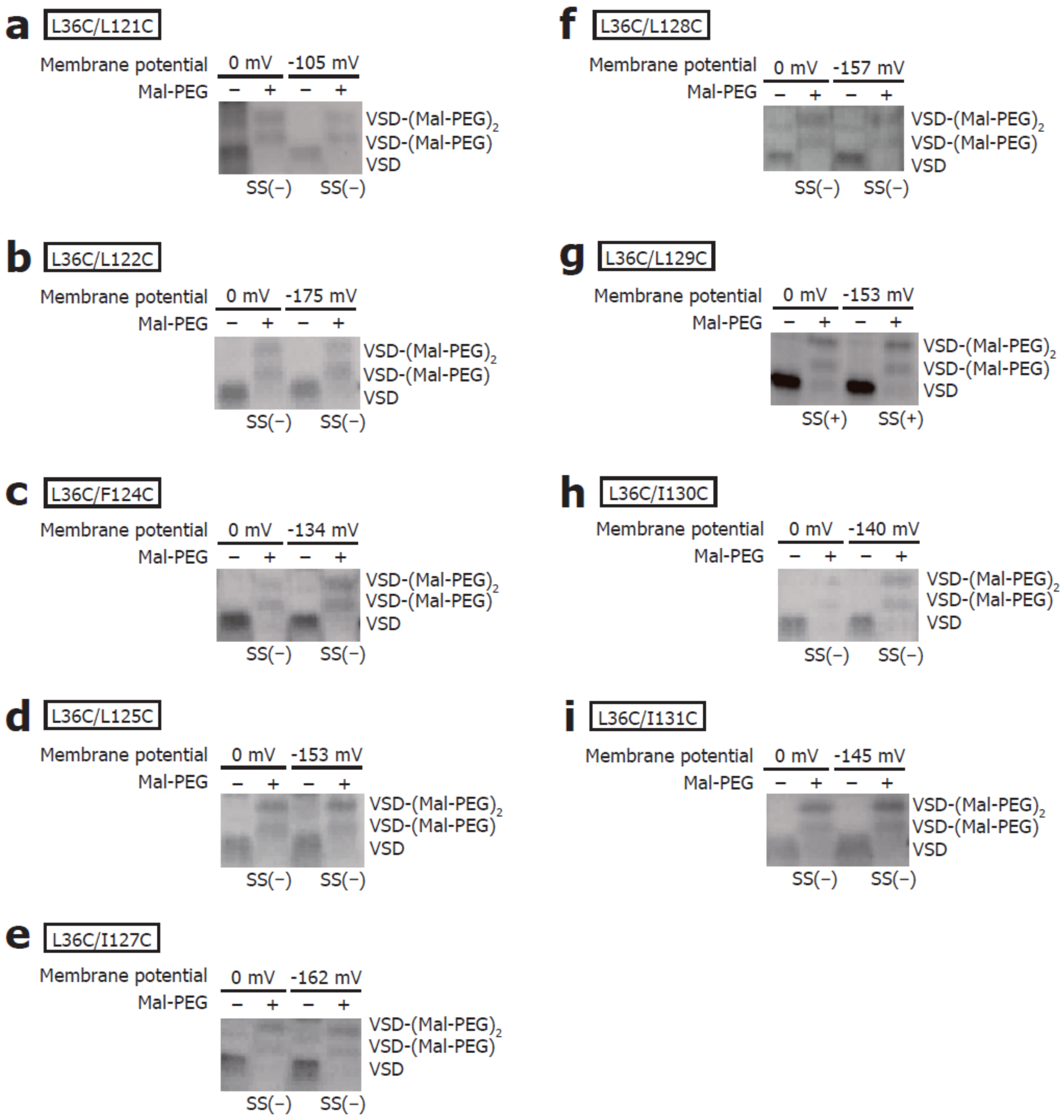

Supplementary Figure S4: The results of the voltage-dependent SS-locking analysis (L36 in S1 was mutated to Cys)

SS(+) stands for the SS-bond formation, as evidenced by the intensity of the unPEGylated band higher than 25% of the total intensity of the PEGylated and unPEGylated bands.

SS(-) stands for no SS-bond formation, as evidenced by the intensity of the unPEGylated band lower than 25% of the total intensity of the PEGylated and unPEGylated bands.

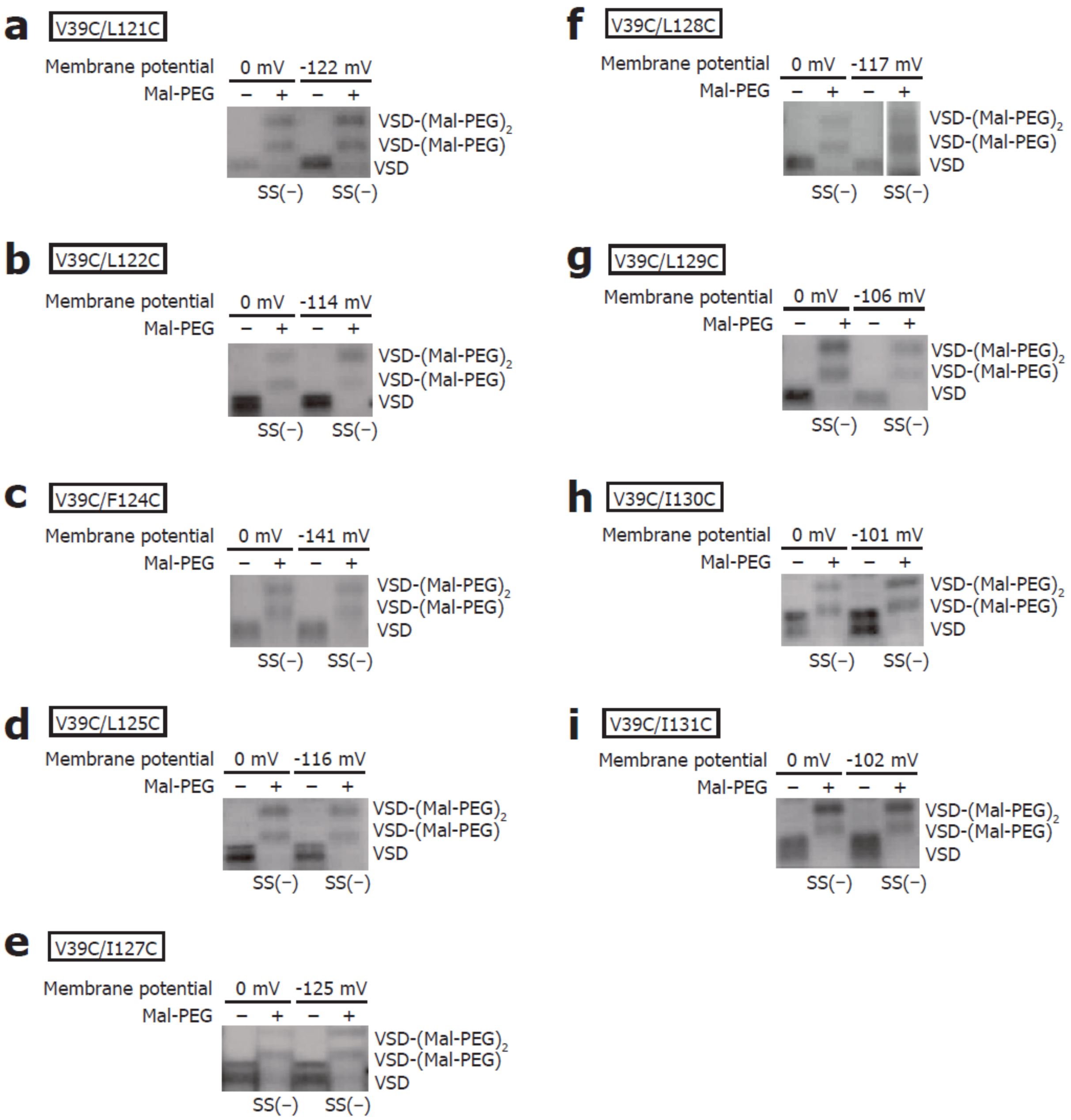

Supplementary Figure S5: The results of the voltage-dependent SS-locking analysis (V39 in S1 was mutated to Cys)

SS(+) stands for the SS-bond formation, as evidenced by the intensity of the unPEGylated band higher than 25% of the total intensity of the PEGylated and unPEGylated bands.

SS(-) stands for no SS-bond formation, as evidenced by the intensity of the unPEGylated band lower than 25% of the total intensity of the PEGylated and unPEGylated bands.

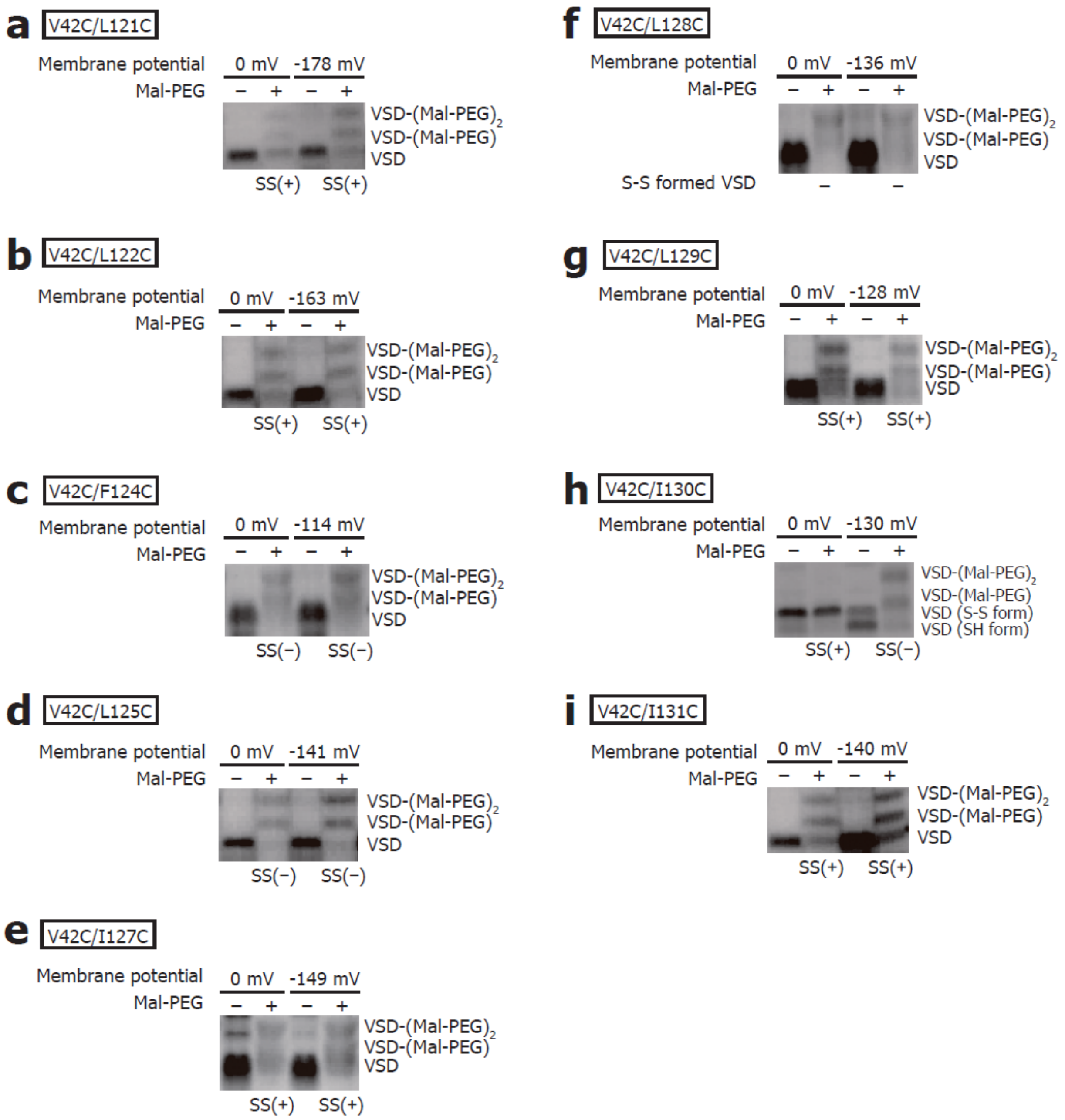

Supplementary Figure S6: The results of the voltage-dependent SS-locking analysis (V42 in S1 was mutated to Cys)

SS(+) stands for the SS-bond formation, as evidenced by the intensity of the unPEGylated band higher than 25% of the total intensity of the PEGylated and unPEGylated bands.

SS(-) stands for no SS-bond formation, as evidenced by the intensity of the unPEGylated band lower than 25% of the total intensity of the PEGylated and unPEGylated bands.

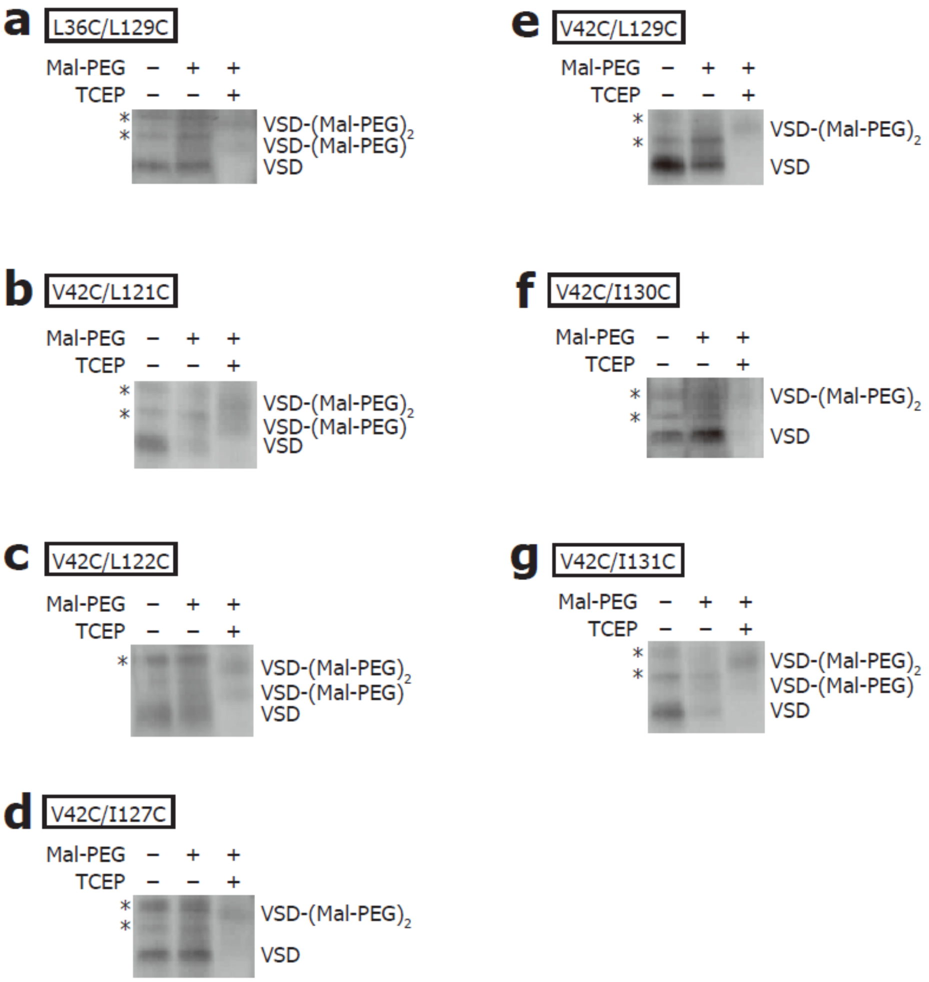

Supplementary Figure S7: Confirmation of the SS-bond formation

In supplementary Figs. S3-S6, SS-bond formation was detected as unPEGylated band. However, the unPEGylated band might be due to oxidation of a thiol group to a sulfino or sulfonic group without the formation of the intramolecular SS-bond. In order to investigate whether the VSD was protected from PEGylation by the SS-bond or not, we reduced SS-bonds after CuP oxidation with TCEP, followed by Mal-PEG modification.

Left lane: sample after oxidation

Middle lane: sample with mal-PEG modification after oxidation

Right lane: sample with reduction and mal-PEG modification after oxidation

UnPEGylated bands were detected in the middle lane, but disappeared in the right lane. These results indicate that the samples before reduction were protected from PEGylation by the intramolecular SS-bond formation. Asterisks indicate the bands of impurity.
